# Knowledge and implementation behavior towards mitigation initiatives of climate change: Community settings approach of Bangladesh context followed cross-sectional design

**DOI:** 10.1371/journal.pone.0307898

**Published:** 2024-08-30

**Authors:** Bilkis Banu, Nasrin Akter, Nusrat Hossain Sheba, Sujana Haque Chowdhury

**Affiliations:** Department of Public Health, Northern University Bangladesh, Dhaka, Bangladesh; Bangladesh Agricultural University, BANGLADESH

## Abstract

Bangladesh experiences different types of natural disasters almost every year which adversely affect human health. It is very essential to identify knowledge and implementation behavior as mitigation initiatives towards climate change in community settings of Bangladesh. This study was designed to explore this issue. It was an analytical type of cross-sectional study which was conducted among 450 adult people residing in Barisal district of Bangladesh. Data were collected through face-to-face interviews using semi-structured questionnaire included socio-demographic information, knowledge and implementation behavior regarding mitigation initiatives towards climate change. Bivariate and multivariate techniques were adopted to analyze the data. The outcome reflected that a large proportion of the respondents had poor knowledge (55.1%) and poor implementation behavior (52.0%) on mitigation initiatives towards climate change. Poor knowledge was significantly more prominent among the people who were male (AOR = 1.56), Muslim (AOR = 2.55), respondents with >4 family members (AOR = 1.91) and with >3 children (AOR = 1.64) showed higher odds of poor knowledge. Poor implementation behavior was found significantly more leading among the female (AOR = 2.91), service-holder (AOR = 1.92) participants having higher monthly family incomes (AOR = 2.91), who had <1 child (AOR = 2.70), belonging ≤4 number of family members (AOR = 30.09). An alarming proportion of poor knowledge and implementation behavior were found regarding mitigation initiatives towards climate change in community settings of Bangladesh. Concerning demographic major predictors, it is essential to plan and implement sustainable and comprehensive health promotional program on climate change mitigation throughout the country.

## Introduction

Severe weather events and accompanying calamities are a global manifestation of climate change(CC), which is currently a major issue [1]. The average worldwide temperature in 2022 was approximately 0.86°C higher than the average of 13.9°C for the 20th century [1, 2]. Moreover, there has been a global shift in the precipitation pattern [1]. These types of environmental degradation and (CC) pose related risks to the planet’s future [1, 3]. There will be catastrophic effects on communities worldwide as a result of the anticipated increase in the frequency and severity of extreme weather incidence [4–6]. South Asia is particularly vulnerable due to a unique combination of socioeconomic, political, and geographical circumstances which include this CC. It’s ironic that South Asian nations, despite contributing the least to global greenhouse gas emissions, are among the most severely impacted [4, 7]. Numerous weather extremes and climate change induced by humans are already being felt in every part of the world. This has resulted in numerous negative effects, losses, and harm to both people and the environment [8]. Research suggests that public opinion on CC and its health impacts may influence policies addressing CC related health issues [9, 10]. Some research indicates that lay people’s perceptions about CC’s causes and effects were obscured [10–13]. These misconceptions often instill anxiety regarding the potential repercussions of CC [10, 14]. To strengthen resilience among the most vulnerable groups, however, government education and environmental change management are crucial [10, 15]. Understanding CC is a prerequisite for people to adjust accordingly [10]. The world’s most varied climate regimes and ecosystems are found in South Asia [16–18]. Bangladesh is regarded as one of the world’s most climate-vulnerable nations [19]. The average temperature has been increasing at a pace of 0.20°C per ten years. Despite making up less than 0.48% of all emissions worldwide, the nation is nonetheless affected by all the negative effects of CC [19]. It was also projected that annual rainfall would increase by 153 mm between 2011 and 2020 [17, 20]. Among all the external forces, the largest threat to Bangladesh is Sea Level Rise (SLR), which is accountable for the country’s 5.8%–9.1% decrease in rice production. The nation is extremely vulnerable to the effects of climate change, which include frequent floods, tropical storms, landslides, and coastal erosion that result in significant loss of life and property [21, 22]. Numerous variables that drive migration will be impacted by CC, which will affect the most vulnerable communities [17, 23]. The effects of CC pose an even greater economic risk in developing countries like Bangladesh, where agriculture plays a central role in the economy by providing income and subsistence to a large segment of society, especially for nations consisting of agro-zones susceptible to submersion [18]. The first groups of people to experience the direct or indirect effects of CC are indigenous peoples. They rely heavily on the resources found in their immediate surroundings, especially the forests [16]. Thus, to meet global mitigation and adaptation targets, a concerted international effort involving funding, technological transfer, and capacity building is needed [17]. Context-specific adaptation tactics vary across time, from place to place, and even within unique societies [24–26]. These techniques are essential for assisting the surrounding communities in adjusting to climate fluctuations and extreme weather [24, 27–31]. The Intergovernmental Panel on Climate Change (IPCC) of the United Nations has centered discussions on developing adaption strategies around local knowledge of adaptation [24, 32]. Another recurrent requirement of this period is strong, immediate action to limit anthropogenic greenhouse gas emissions; otherwise, global warming and shifting climatic patterns will only worsen [2]. The contribution of quickly developing nations to mitigating CC is gaining attention [21]. Given the serious climate risks, Bangladesh must explore and understand adaptation techniques to mitigate negative impacts, especially for marginalized riverine people. Farmers are adapting by growing saline-tolerant rice, experimenting with new varieties and planting dates, converting paddies to fish production, and practicing crop rotation [17]. However, in light of the growing hazards and constraints posed by CC, community-based adaptation strategies are beginning to get substantial worldwide attention [33, 34]. The awareness of local contexts, priorities, and the needs of the impacted local people is the only foundation for the profound adaptation measure at the community level, which is subsequently built on ongoing local-based initiatives [33, 35]. However, Bangladesh has conducted very little research on adaptability [24]. Understanding CC is essential for the people for effective adaptation. However, very few studies focus on knowledge and implementation behavior related to CC mitigation, highlighting the need for more research in this area. Therefore, the results of this study aimed to provide valuable insights on this crucial issue within Bangladeshi communities.

## Methods

### Study design

A quantitative cross-sectional study was carried out followed analytical approach due to gathered data were from a group of subjects at only one point in time and data evaluation need to be done critically. Semi-structured data were collected in this study during January to June 2023 for obtaining the information on socio-demographic information, knowledge and implementation behavior as mitigation initiatives towards climate change among adult people residing in Barisal district of Bangladesh.

### Study participants, sample size and sampling

This study considered a total of 450 adult people residing in Barisal district of Bangladesh. Quantitative information of this study was collected from the respondents’ identified as the adult (aged 18 to 49 years) residents’ habitat in the study place, signified as household head and provided their consent to participate in this study.

Initially a potential standard sample size was assumed as 299 calculated by using the formula “n = ‘Z^2^pq/d^2^” where Z (standard normal deviate) considered as 1.96; p (knowledge on climate change 54.2%identified in a previous study) was considered as 0.54 [10] and margin of error was considered as 0.05. With a minimum calculated sample 381, an additional 10% was added as cushion to take into account non-response and 8% was added considering data cleaning and initial management and the final samples were 450.

A random sampling technique was used in this study to generalize the findings. A total number of 9.3 million populations of Barisal division were considered as study population amongst the eight divisions (Dhaka, Chittagong, Rajshahi, Khulna, Rangpur, Mymensingh, Sylhet and Barisal) of Bangladesh [36]. Barisal division was selected through multi-stage random sampling as study place. In addition, Barisal is a coastal region, bounded by the Bay of Bengal on the south and one of the most vulnerable zones for climate-induced disasters [37]. In line of this, Barisal district was considered for this study under the division concerning as divisional district. This study considered three wards (optional sub-division of city) which were randomly selected from the 30 wards under the City Corporation (urban level administrative body) of Barisal district followed a list provided by the local administrative authority. 150 households were randomly selected from the household list of each ward and 450 households were finally selected for the study. Head of each household were considered as the participants of this study.

### Data collection

Quantitative data was collected from the community people by using a pre-tested and semi-structured questionnaire through the face-to-face interview method. Respondents were recruited in the study and interviewed according to their pre-given schedule (as rural people usually occupied with different manual labors) during the period of February to April 2023. Data collectors were well trained and closely monitored by the research team for avoiding interviewer bias. The interviewer took only 10 to 15 minutes to complete the survey. All authors had access to the collection and preserving participants’ information during or after data collection. The survey was administered in the Bengali language with the utmost support of the local administrative authority.

### Ethical considerations

This study was approved by the Ethical Review Committee of the Department of Public Health of Northern University Bangladesh (NUB/DPH/EC/2023/24) and conformed to the Declaration of Helsinki. Participation of the respondents was anonymous and voluntary. Written informed consent was taken from the respondents at the beginning of the survey, participants could withdraw from the survey at any time and their autonomy has been ensured.

### Questionnaire design

The questionnaire, developed from variables found in published literature, was reviewed by two independent researchers and pre-tested on 10 respondents. The pre-test responses were used to refine the questionnaire. The need for entire information led to the use of a semi-structured questionnaire. The pivotal components of the questionnaire were: (i) Socio-demographic information: gender, religion, family size, number of children, occupation, monthly family income, living duration; (ii) Knowledge on climate change: definition of climate change, causes of climate change, types of natural changes, types of natural disasters, types of changes occur on sea/ coast, health problems due to climate change, livelihood problems, prevention by environmental change, prevention by infrastructure related change, prevention by livelihood change; (iii) Behavior on prevention and mitigation of climate change: tree plantation, cutting down trees, involved with cultivation and home gardening, having room with ≤1 window, cleaning of outside surface and drainage of home, using solar system, using biogas, disposal of household waste in proper place, using paper/ cloth bags to carry goods, electric switch turn off habit, preparation and management of natural disaster.

### Data analysis

Collected data was checked and analyzed employing the Statistical Package for the Social Sciences (SPSS) software(version 26.0). Study characteristics were subjected to descriptive statistics (frequency and proportions) to summarize the obtained data.

The components of knowledge and implementation behavior as mitigation initiatives towards climate change were scored and categorized followed mid values of the percentage scores as cut points. Relevant continuous data were also categorized followed similar ways [38]. A multinomial logistic regression analysis was performed followed by a modeling procedure to adjust the confounders. This analysis was considered a backward elimination process, including pre-specified variables identified as significant association with dependent variable by chi-square testi.e. gender, religion, family size, number of children, occupation, monthly family income, living duration. Odds Ratios with 95% confidence intervals with respect to knowledge and behavior regarding prevention and mitigation of climate change (poor and moderate/ good) were calculated for the specified exposures.

## Results

### Participant’s characteristics

A total of 450 respondents were considered in this study, with the majority aged more than 20 years old (87.3%) and Muslims (94%), 68.4% of whom were female and nearly half (43.8%) had up to SSC educational qualification. By occupation, nearly half of the respondents (45.6%, n = 205/450) were housewives; 20.9% were service holders, and 16.7% were students. Majorities (66.9%, n = 301/450) of the respondents had less than or equal to four family members in their family and belonged to an extended family (56.7%). Most respondents have been living for less than or equal to 20 years (62.7%, n = 282) in their own residences (85.3%). Moreover, the majority (67.6%, n = 304/450) had less than 15.04 USD in monthly household income, representing a sign of low socioeconomic condition (Table 2).

### Insights of knowledge and behavior regarding prevention and mitigation of climate change among the respondents

The prime outcome of this study is delineated in Table 1. It is revealed that most of the respondents had poor knowledge (55.1%) and behavior (52.0%) regarding the prevention and mitigation of climate change. Surprisingly, it is observed that a good level of knowledge is significantly (COR/p = 1.99/0.01) associated with the poor behavior of the study subjects, which indicates a need for promotional facilities for the proper practice of prevention and mitigation of climate change issues (Table 1).

**Table 1:** Distribution of respondent’s knowledge according to their behavior regarding prevention and mitigation of climate change (n = 450).

Knowledge	Number of participants, n (%)	Behavior		COR/ p
		Good, n (%)	Poor, n (%)	
**Good**	202 (44.9)	40 (8.9)	73 (16.2)	1.99/ 0.01*
**Poor**	248 (55.1)	176 (39.1)	161 (35.8)	
**Total**	450 (100%)	216 (48.0%)	234 (52.0%)	

*Statistically Significant

Data are presented as frequency (n) and percentage (%); Logistic Regression Analysis was used to identify the predictors. *Statistical significance at p-value ≤0.05; reference category was considered for the level of knowledge and behavior as "Good."

**Fig 1:**
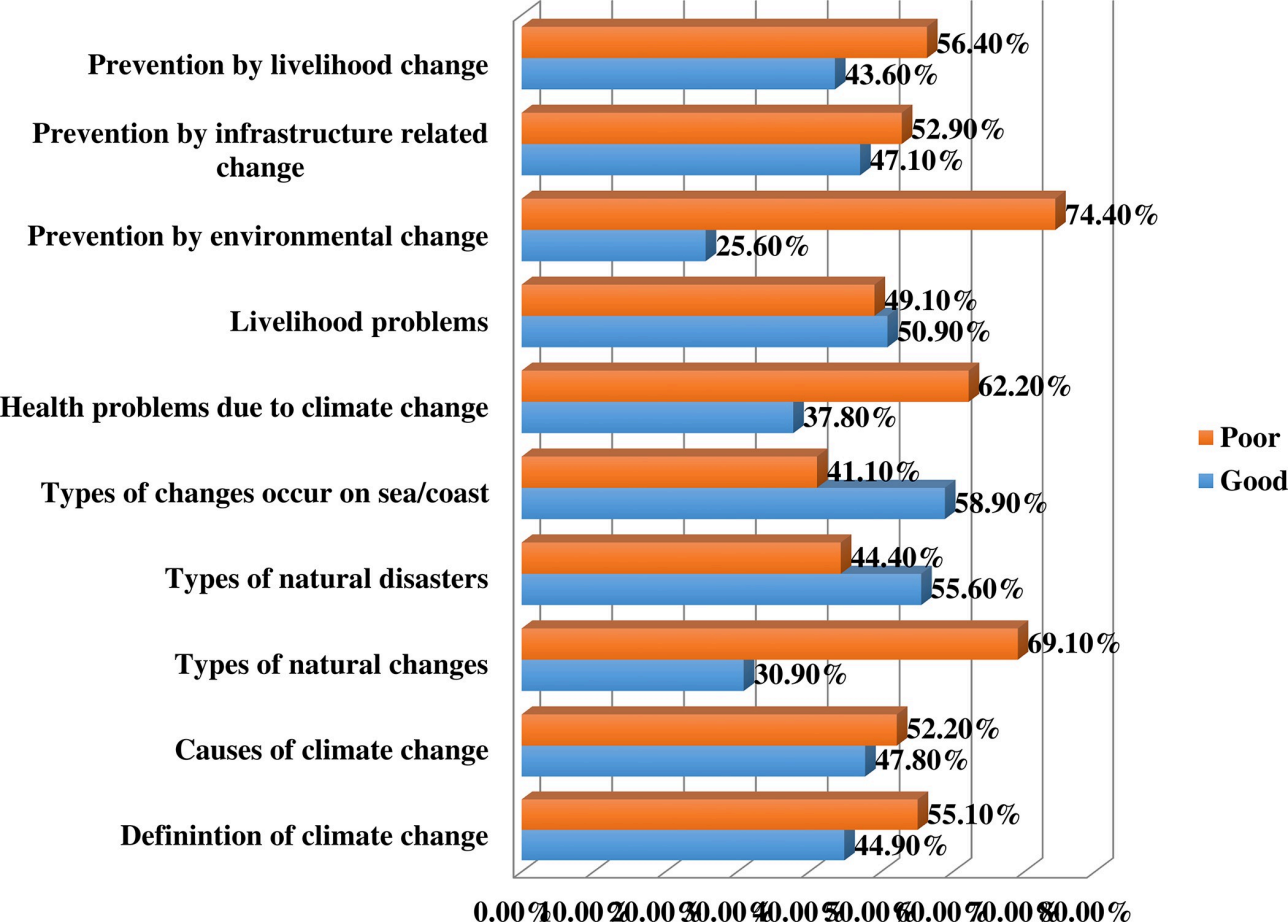
This is the insights of knowledge on climate change (n = 450).

To assess the level of knowledge, we considered several basic components associated with the prevention and mitigation of climate change that are pivotal to combating its consequences. Likewise, components include the definition and causes of climate change, types of changes in nature as well as sea/coastal areas, types of natural disasters, livelihood problems, and health problems due to climate change, prevention by infrastructure-related changes, environmental changes, and prevention of livelihood change. The level of knowledge for all the individual components was found to be poor, mostly except for the knowledge of livelihood problems (good knowledge = 50.90%), types of changes in sea/coastal areas (good knowledge = 58.90%), and types of natural disasters (good knowledge = 55.60%). Moreover, significantly higher levels of poor knowledge were found for the components ‘prevention by environmental change’ (74.40%), ‘types of natural changes’ (69.10%), health problems due to climate change (62.20%), and prevention by livelihood changes (56.40%)which had major contribution for the overall poor knowledge of the subjects. In line with that, preventive approach regarding poor knowledge of “prevention by environmental change” (74.4%) and “types of natural changes” (69.1%) could have significant impacts on effective climate change mitigation practice (Fig 1).

**Fig 2:**
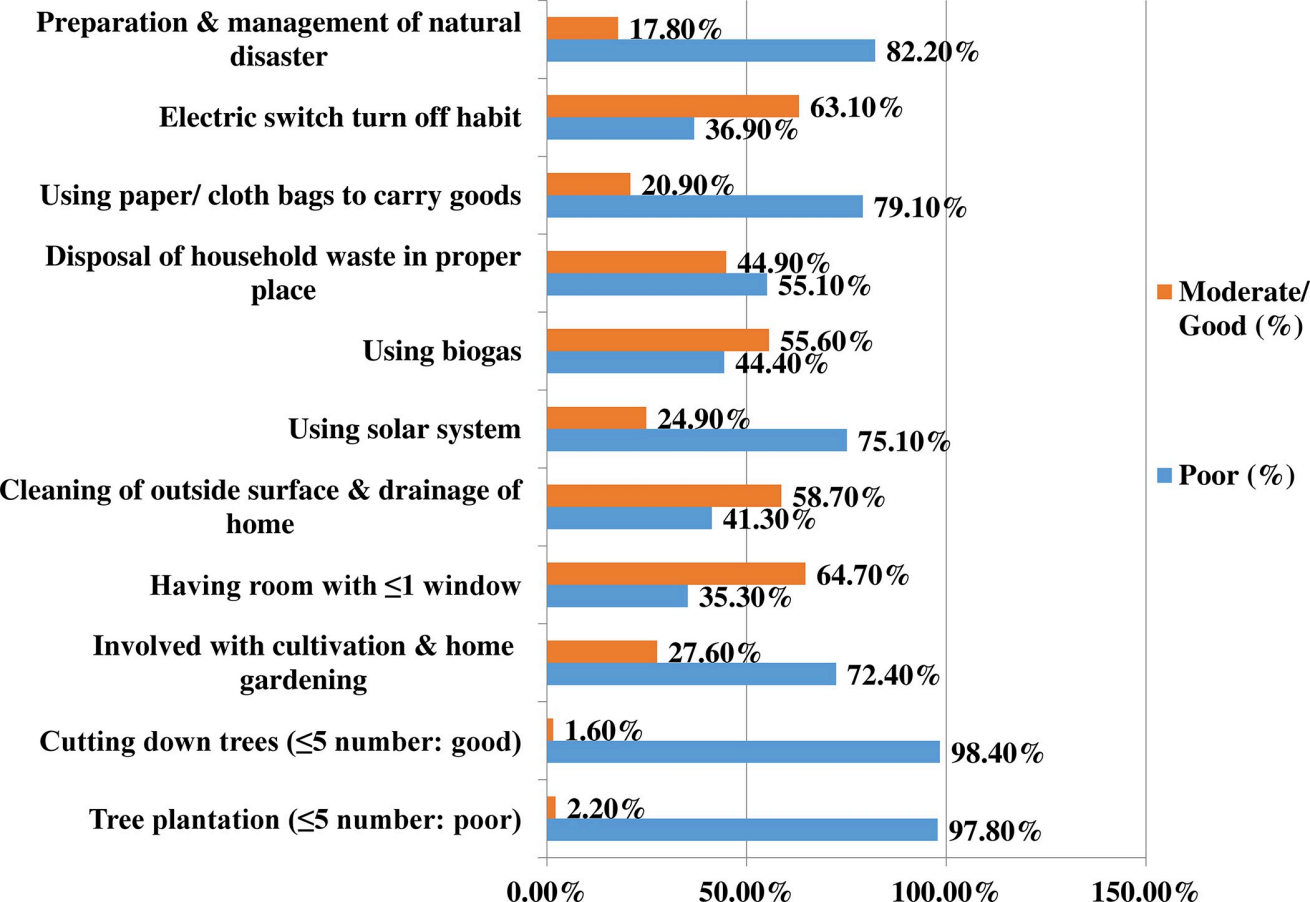
This is the insights of behavior on climate change (n = 450).

On the other hand, the behavior level of climate change prevention and mitigation behavior was assessed, considering some significant components that influence human behavior toward the climate change mitigation and adaptation milestone. Components include tree plantation, cutting down trees, being involved with cultivation and home gardening, having rooms with ≤1 window, cleaning of outside surface and drainage of home, using solar system, using solar system, using biogas, disposal of household waste in the proper place, using paper or cloth bags to carry goods, electric switch turn off habit and preparation and management of natural disaster. Significant good behavior was found mostly for the components such as cutting down trees (≤5 number) (98.40%), tree plantation (97.80%), using the solar system (75.10%), being involved with cultivation and gardening (72.40%), using paper and cloth bags to carry goods (79.10%), and being prepared for the management of natural disaster (82.20%). In the contrary, most of the respondents had poor behavior regarding electric switch turn off habit (63.10%), did not have support to use biogas (56.60%), had rooms with less than or equal one window (64.7%), and did not clean outside area and drainage of their home (58.70%). The poor behavioral status indicates poor policy related support tends to make their practice poor in such components though they had good practice in other components. This indicates the need for comprehensive promotional approach towards climate change mitigation (Fig 2).

### Respondent’s characteristics associated with the level of knowledge and behavior regarding prevention and mitigation of climate change

Multivariate cross-tabulation analysis of the study revealed the significant underlying characteristics associated with the respondents’ knowledge and behavior regarding climate change prevention and mitigation. Likewise, the knowledge of the respondents was found to be significantly associated with gender (χ2/p = 5.10/0.02), religion (χ2/p = 10.92/0.01), family size (χ2/p = 10.17/0.01), number of children (χ2/p = 3.90/0.04), family type (χ2/p = 4.84/0.02), living duration (χ2/p = 6.32/0.01), and types of residence (χ2/p = 8.39/0.01) of the respondents. Then again, moving toward the behavior study also revealed some significant criteria associated with it. For instance, age (χ2/p = 8.64/0.01), gender (χ2/p = 10.34/0.01), religion (χ2/p = 18.96/0.01), education (χ2/p = 7.71/0.05), occupation (χ2/p = 11.16/0.03), family size (χ2/p = 210.42/0.01), number of children (χ2/p = 13.16/0.01), family type (χ2/p = 45.95/0.01), living duration (χ2/p = 71.54/0.01), types of residence (χ2/p = 43.33/0.01) and monthly household income (χ2/p = 52.88/0.01) of the respondents were found to be significantly associated with the poor behavior of the respondents (Table 2).

**Table 2:** Underlying demographic characteristics associated with the level of knowledge and behavior regarding the prevention of climate change among the respondents (n = 450).

Characteristics	Number of participants, n (%)	Knowledge on prevention of climate change			Behavior on prevention of climate change		
		Good, n (%)	Poor, n (%)	χ^2^/p-value (≤0.05)	Good, n (%)	Poor, n (%)	χ^2^/p-value (≤0.05)
**Age (In years)**	** **	** **	** **	** **	** **	** **	** **
≤20	57 (12.7)	20 (4.4)	37 (8.2)	3.45/0.06	17 (3.8)	40 (8.9)	8.64/0.01*
>20	393 (87.3)	93 (20.7)	300 (66.7)		199 (44.2)	194 (43.1)	
**Gender**	** **	** **	** **	** **	** **	** **	** **
Male	142 (31.6)	26 (5.8)	116 (25.8)	5.10/0.02*	84 (18.7)	58 (12.9)	10.34/0.01*
Female	308 (68.4)	87 (19.3)	221 (49.1)		132 (29.3)	176 (39.1)	
**Religion**	** **	** **	** **	** **	** **	** **	** **
Muslim	423 (94.0)	99 (22.0)	324 (72.0)	10.92/0.01*	214 (47.6)	209 (46.4)	18.96/0.01*
Non-Muslim	27 (6.0)	14 (3.1)	13 (2.9)		2 (0.4)	25 (5.6)	
**Education (In years)**	** **	** **	** **	** **	** **	** **	** **
Illiterate	116 (25.8)	30 (6.7)	86 (19.1)	1.58/0.66	57 (12.7)	59 (13.1)	7.71/0.05*
Up to Primary	87 (19.3)	18 (4.0)	69 (15.3)		35 (7.8)	52 (11.6)	
Up to SSC	197 (43.8)	50 (11.1)	147 (32.7)		106 (23.6)	91 (20.2)	
HSC or Above	50 (11.1)	15 (3.3)	35 (7.8)		18 (4.0)	32 (7.1)	
**Occupation**	** **	** **	** **	** **	** **	** **	** **
Service	94 (20.9)	25 (5.6)	69 (15.3)	5.17/0.27	32 (7.1)	62 (13.8)	11.16/0.03*
Day labor/Farmer	37 (8.2)	8 (1.8)	29 (6.4)		16 (3.6)	21 (4.7)	
Business	39 (8.7)	6 (1.3)	33 (7.3)		23 (5.1)	16 (3.6)	
Housewife	205 (45.6)	49 (10.9)	156 (34.7)		106 (23.6)	99 (22.0)	
Student/Unemployed/Retired	75 (16.7)	25 (5.6)	50 (11.1)		39 (8.7)	36 (8.0)	
**Family size (In numbers)**	** **	** **	** **	** **	** **	** **	** **
≤4	282 (62.7)	85 (18.9)	197 (43.8)	10.17/0.01*	61 (13.6)	221 (49.1)	210.42/0.01*
>4	168 (37.3)	28 (6.2)	140 (31.1)		155 (34.4)	13 (2.9)	
**Number of children (In numbers)**	** **	** **	** **	** **	** **	** **	** **
One	109 (24.2)	37 (8.2)	72 (16.0)	3.90/0.04*	41 (9.1)	68 (15.1)	13.16/0.01*
Two	74 (16.4)	15 (3.3)	59 (13.1)		48 (10.7)	26 (5.8)	
Three or More	267 (59.3)	61 (13.6)	205 (45.8)		127 (28.2)	140 (31.1)	
**Family type**	** **	** **	** **	** **	** **	** **	** **
Living alone/ Nuclear	195 (43.3)	59 (13.1)	136 (30.2)	4.84/0.02*	58 (12.9)	137 (30.4)	45.95/0.01*
Extended	255 (56.7)	54 (12.0)	201 (44.7)		158 (35.1)	97 (21.6)	
**Living duration (In years)**	** **	** **	** **	** **	** **	** **	** **
≤20	282 (62.7)	82 (18.2)	200 (44.4)	6.32/0.01*	92 (20.4)	190 (42.2)	71.54/0.01*
>20	168 (37.3)	31 (6.9)	137 (30.4)		124 (27.6)	44 (9.8)	
**Types of residence**	** **	** **	** **	** **	** **	** **	** **
Own	384 (85.3)	87 (19.3)	297 (66.0)	8.39/0.01*	209 (46.4)	175 (38.9)	43.33/0.01*
Rental/Govt.	66 (14.7)	26 (5.8)	40 (8.9)		7 (1.6)	59 (13.1)	
**Monthly family income (In USD)**		** **	** **	** **	** **	** **	** **
≤154.88	304 (67.6)	70 (15.6)	234 (52.0)	2.17/0.14	182 (40.4)	122 (27.1)	52.88/0.01*
>154.88	146 (32.4)	43 (9.6)	103 (22.9)		34 (7.6)	112 (24.9)	

Data are presented as frequency (n) and percentage (%); the Chi-square test was used to observe the association, *Statistical significance at p-value ≤0.05.

### Predictors associated with the level of knowledge and behavior regarding prevention and mitigation of climate change

**Fig 3:**
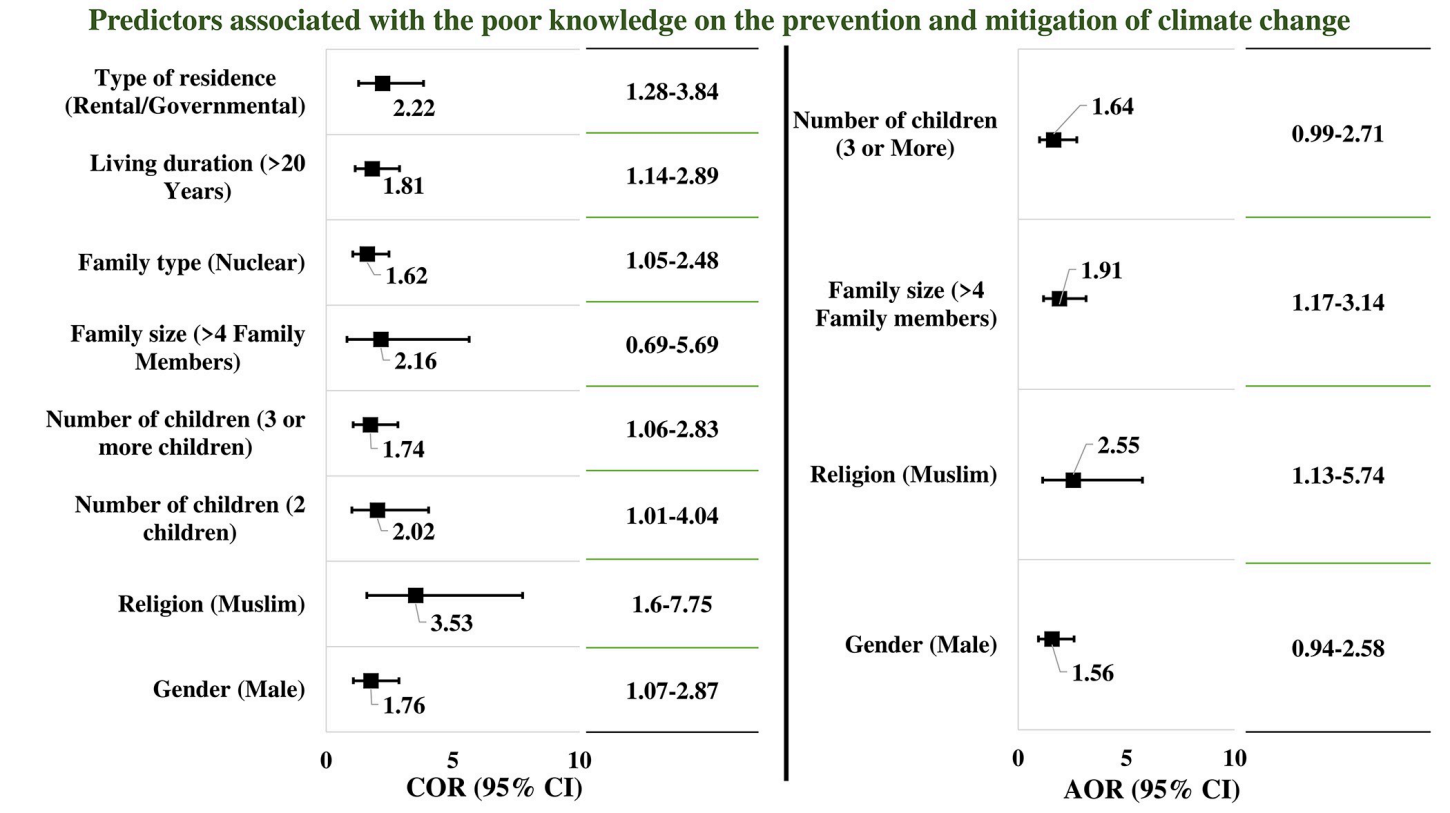
This is the predictors influencing the level of knowledge regarding prevention and mitigation of climate change (n = 450).

Regression analysis of the study revealed significant predictors associated with the level of knowledge and behavior regarding the prevention and mitigation of climate change. From the insights of the level of knowledge, the study revealed that significant poor knowledge was found among the male (COR/p = 1.76/0.02, 95% CI = 1.07–2.87) Muslim (COR/p = 3.53/0.01, 95% CI: 1.60–7.75) respondents who had 2 or more children (2 children: COR/p = 2.02/0.04, 95% CI: 1.01–4.04; 3 or more children: COR/p = 1.74/0.03, 95% CI: 1.06–2.83) and a larger number of family members (>4 members: COR/p = 2.16/0.01, 95% CI: 1.06–2.83), who were belonging to a nuclear family (COR/p = 1.62/0.03, 95% CI: 1.05–2.48), and lived in a rental/governmental house (COR/p = 1.81/0.01, 95% CI: 1.14–2.89) for more than 20 years (COR/p = 2.22/0.01, 95% CI: 1.28–3.84). Final significant predictors were found once the adjusted modeling was done and confounders were eliminated through the backward elimination procedure. As significant predictors of poor knowledge of climate change prevention and mitigation, the study revealed that male (AOR/p = 1.56/0.06, 95% CI: 0.94–2.58) Muslim (AOR/p = 2.55/0.03, 95% CI: 1.13–5.74) respondents with larger family members (AOR/p = 1.91/0.01, 95% CI: 1.17–3.14) and 3 or more children (AOR/p = 1.64/0.05, 95% CI: 0.99–2.71) showed higher odds of poor knowledge regarding the prevention and mitigation of climate change (Fig 3).

Fig 3 indicated statistically significant predictor is considered at p ≤0.05. Reference category was considered for the level of knowledge as "Good". Reference category for gender is female, for religion is non-Muslim, for number of children is one children, for family size is ≤4 family members for family type is extended family, for living duration is ≤20 years, and lastly for type of residence is own.

**Fig 4:**
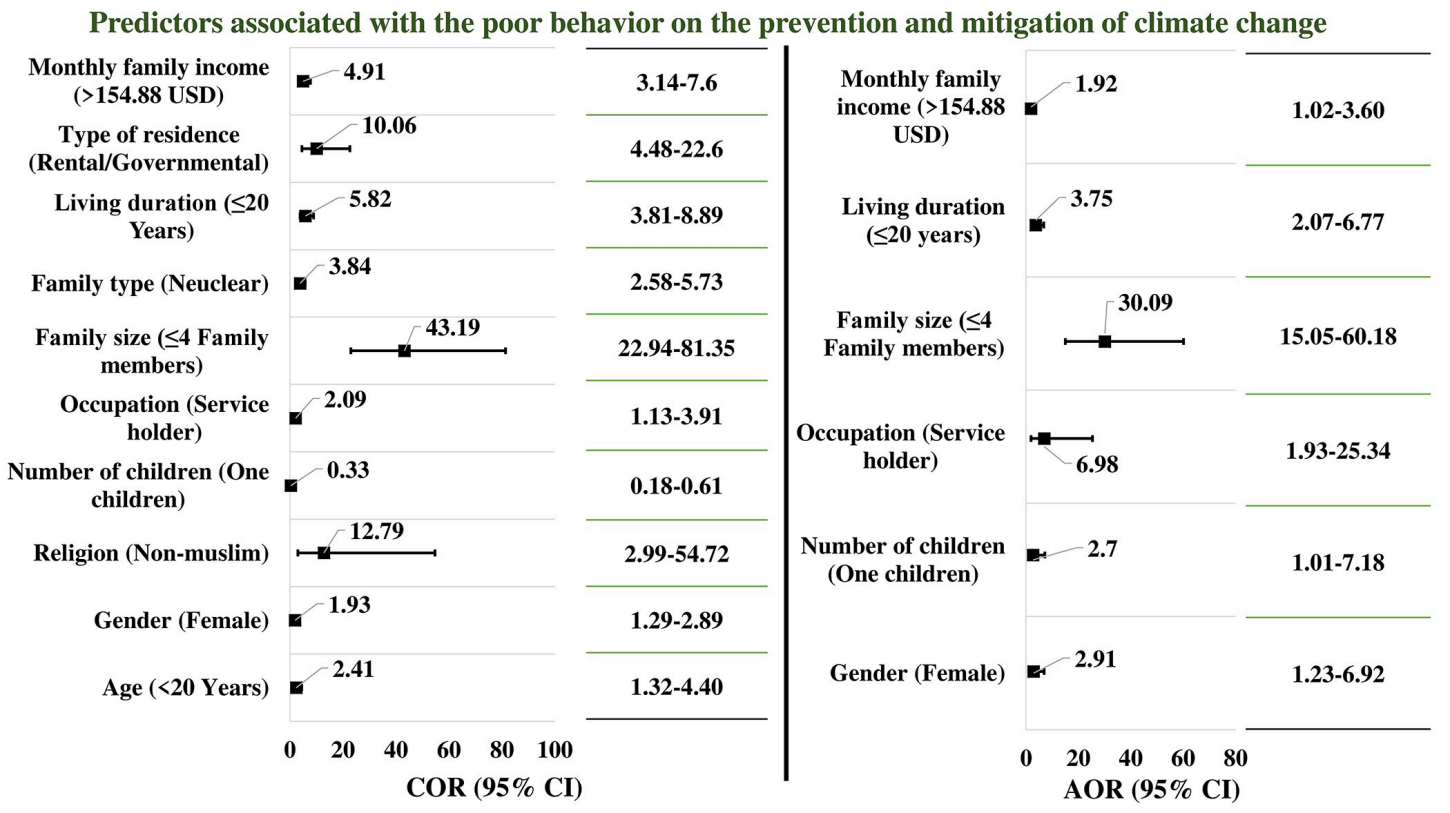
This is the predictors influencing the behavior regarding prevention and mitigation of climate change (n = 450).

On the other side, the study also revealed some significant predictors influencing the poor behavior of the respondents toward climate change mitigation through performing regression analysis, including model adjustment and elimination of confounders. Significant poor behavior in the prevention of climate change issues was found among the respondents with the basic criteria contrary to the predictors of poor knowledge. Female (COR/p = 1.93/0.01; AOR/p = 2.91/0.02) and service-holder (COR/p = 2.09/0.02; AOR/p = 1.92/0.01) respondents with higher monthly family incomes (>154.88 USD: COR/p = 4.91/0.01; AOR/p = 2.91/0.04) who had only one child (AOR/p = 2.70/0.05) with a smaller number of family members (≤4 family members: COR/p = 43.19/0.01; AOR/p = 30.09/0.01) were found to have higher odds of poor behavior to mitigate climate change issues (Fig 4).

Fig 4 indicated statistically significant predictor is considered at p ≤0.05. Reference category was considered for the level of behavior as "Good". Reference category for age is ≤20 years, for gender is male, for religion is Muslim, for number of children is three or more children, for occupation is Student/Unemployed/Retired, for family size is >4 family members for family type is extended family, for living duration is >20 years, for type of residence is own, and lastly for monthly family income is ≤154.88.

## Discussions

This study provides a comprehensive analysis of mitigation measures for climate change implemented in community settings of Bangladesh. The findings revealed that more than half (poor knowledge: 55.1%, poor implementation behavior: 52.0%) of the community people are evident to have a poor state of mitigation measures. These findings in consistent with another study concerning vulnerable groups of community asserting majority of the participants (54.2%) had some knowledge about CC whereas nearly half of the subjects (45.8%) had poor knowledge [10]. A good level of knowledge was observed related to livelihood problems, types of changes in sea/coastal areas, and types of natural disasters. Whereas, higher levels of poor knowledge were found for the components like prevention by environmental change, types of natural changes, health problems due to climate change, and prevention by livelihood changes. Another study from Grenada, Europe found that among the student group, fishermen, and community people of Grenada the community residents were the least knowledgeable about climate change [39]. Nonetheless, it was interesting to find that instead of having good knowledge respondents significantly (p = 0.01) were associated with poor implementation behavior suggesting the need for educational resources to support proper prevention and mitigation of CC issues. This finding is similar to a study, which stated that understanding climate change could not change the implementation behaviors. The study also stated that people’s environmental knowledge is imprecise and influenced by cultural, emotional, and cognitive variables, affecting how they interpret information about CC and how motivated they are to act [40]. In this study several basic components of knowledge on the prevention and mitigation of CC were analyzed where mixed responses were noted. To sketch the behavior level of climate change; community people’s prevention and mitigation behavior level was assessed which as well showed mixed approach. In this study, some acts such as tree plantation, using the solar system, discouraging cutting down trees, being involved with cultivation and gardening, using paper and cloth bags to carry goods, and being prepared for the management of natural disasters were in good practice. But in some crucial components, notable bad behaviors were also discovered like turning off electric switches, biogas use, having rooms with less than or equal to one window, did not cleaning outside areas and drainage of their home. No study was found pointing out why these good practices are prevalent and why these bad practices are lacking. On the other hand, a study finding in Zambia identified the causes under four major themes: dispositional, institutional, situational, and technical barriers, which are responsible for adopting the biogas [41].

Concerning basic responsible characteristics associated with the level of knowledge and behavior regarding the prevention of climate change among the respondents it was observed that few relatable features like gender, religion, family size and type with number of children, living duration, and resident type were associated with both poor knowledge and behavior on climate change. However, the age of respondents, along with their education, employment status, and monthly income, significantly influences their behavior towards CC. It seems like behaving or practicing good acts to save climate and environment partially depends upon respondents’ daily scraps. Lower education and income levels correlate with reduced sense of responsibility towards nature, as it may not be seen as a priority in meeting basic needs within communities. But this present finding correlates with a study in an Egypt setting suggesting that, aged of >30 years, married Egyptian participants, urban residents, highly educated individuals, and employed individuals got good knowledge of climate change in Egypt [42]. Another study among students of Ghana shows that respondents’ level of education, ethnicity, religion and mother’s occupation got significant association with their knowledge, perception, and attitude on aspects of CC [43]. Furthermore, this supports the idea that higher education and income stability correlate with greater knowledge about CC.

Further investigation into predictors revealed that Muslim male respondents with >3 children and living in families with >4 members showed poor knowledge of climate change. Conversely, female service holders with only one child, low monthly income, and over twenty years of residence had poor engagement in CC mitigation practices. Both predictors give mixed responses with a valid explanation. Male respondents with more burdens in life basically do not pay any attention to knowledge on changed climate. On the other side, female respondents with huge responsibility of family with less income are unable to practice adequate level of mitigation initiatives to save the environment instead of having good knowledge. Very little study found to explore associated predictors regarding this vital issue. A study from Bangladesh suggested that education, sex, and availability of schools in the area were significantly associated with knowledge on CC [10].

This study is subject to several limitations among them the respondents were recruited only from a vulnerable division of Bangladesh leaving the rest of the 7 divisions behind. Therefore, the findings of this study are unlikely to represent all community people across the entire country, thereby impacting the study’s generalizability. For this reason, there is a need to conduct similar type of study on large number of populations among the rest of the divisions. However, these subjects were not significantly different from the study population on their basic criteria or study variables. Another limitation is the cross-sectional nature of this study, which limits causal inferences. In that case the randomized controlled trial would be stronger if conducted. These findings highlight the need to address the fact that we need to build resources solely to educate our community people about the urgency of mitigation initiatives towards climate change as a disaster-prone country. In that case, the study suggests specific types of educational interventions such as community workshops, school programs, or media campaigns regarding climate change. The study also offers policy recommendations for example, policies that could support economically disadvantaged groups in adopting better climate mitigation behaviors.

## Conclusions

The study finding shows that more than one third of the study participants had good implementation behavior despite having poor knowledge. Besides, among our ten components of knowledge, our study participants had poor knowledge regarding the seven components. The higher percentages of poor knowledge were found for the components of prevention by environmental change, types of natural changes, health problems due to climate change, and prevention by livelihood changes. Similarly, they had poor implementation behavior too regarding majority of the components and the higher percentages were found among the components of electric switch turn off habit, did not have support to use biogas, had rooms with less than or equal one window, and did not clean outside area and drainage of their home. Moreover, participants who are male and had larger family member with more children had poor knowledge. On the other hand, female participants had smaller family members with one child had poor implementation behavior. It is strongly advised that more extensive research should be done to investigate the knowledge connected to climate change adaptation that could be useful to researchers and policy makers. Adopting implementation practices is also highly advised, particularly for the female population. In general, the study findings have practical applications for comprehensive community-level interventions in Bangladesh. There is very little information exchanged between the authorities of the central government and the local government institutions (LGIs). Improved integration with LGIs and central government is needed in the implementation stages as they are the leading stakeholders to make policy regarding mitigation initiatives towards climate changein community settings of Bangladesh.

## Supporting information

S1 Data (XLS)
